# Multicenter Observational Study to Evaluate the Diagnostic Value of Sonography in Patients with Chronic Rhinosinusitis

**DOI:** 10.3390/diagnostics12092065

**Published:** 2022-08-26

**Authors:** Alessandro Bozzato, Christoph Arens, Maximilian Linxweiler, Victoria Bozzato, Peter Jecker, Gregor Hilger, Hans-Jürgen Welkoborsky, Johannes Zenk, Lukas Pillong

**Affiliations:** 1 Department for Otorhinolaryngology and Head and Neck Surgery, Saarland University Medical Center, Kirrbergerstraße 100, 66421 Homburg, Germany; 2 Department for Otorhinolaryngology and Head and Neck Surgery, University Hospital Giessen, Klinikstraße 29, 35392 Gießen, Germany; 3 Department of Otorhinolaryngology and Plastic Head and Neck Surgery, Klinikum Bad Salzungen GmbH, Lindigallee 3, 36433 Bad Salzungen, Germany; 4 Department for Otorhinolaryngology and Head and Neck Surgery, Klinikum Stolberg, Jahnsdorfer Straße 7, 09366 Stollberg, Germany; 5 Department for Otorhinolaryngology and Head and Neck Surgery, Klinikum Nordstadt, Haltenhoffstr. 41, 30167 Hannover, Germany; 6 Department for Otorhinolaryngology and Head and Neck Surgery, University Hospital Augsburg, Sauerbruchstr. 6, 86179 Augsburg, Germany

**Keywords:** chronic rhinosinusitis, paranasal sinuses, sonography, computed tomography, ultrasound, A-scan, B-scan, diagnostic imaging

## Abstract

(1) Background: Computed tomography (CT) is considered mandatory for assessing the extent of pathologies in the paranasal sinuses (PNS) in chronic rhinosinusitis (CRS). However, there are few evidence-based data on the value of ultrasound (US) in CRS. This multicenter approach aimed to compare diagnostic imaging modalities in relation to findings during surgery. (2) Methods: 127 patients with CRS were included in this prospective multicenter study. Patients received preoperative US and CT scans. The sensitivity and specificity of CT and US were extrapolated from intraoperative data. (3) Results: CT scans showed the highest sensitivity (97%) and specificity (67%) in assessing CRS. Sensitivities of B-scan US were significantly lower regarding the maxillary sinus (88%), the ethmoid sinus (53%), and the frontal sinus (45%). The highest overall sensitivity was observed for assessing the pathology of the maxillary sinus. (4) Conclusions: We observed high accuracy with CT, confirming its importance in preoperative imaging in CRS. Despite the high US expertise of all investigators and a standardized examination protocol, the validity of CT was significantly higher than US. Ultrasound of the PNS sinuses is applicable in everyday clinical practice but lacks diagnostic accuracy. Nevertheless, it might serve as a complementary hands-on screening tool to directly correlate the clinical findings in patients with PNS disease.

## 1. Introduction

Chronic rhinosinusitis (CRS) is a common disease of the paranasal sinuses (PNS). According to the current criteria [1], CRS is present if two or more of the following clinical symptoms persist for a period of more than 12 weeks: nasal obstruction, anterior and/or posterior nasal secretion, facial pain and/or headache, and olfactory diminution [1]. Endoscopic findings of mucopurulent secretion and evidence of mucosal swelling in the PNS on imaging studies support the diagnosis [2]. Among the imaging examination methods, computed tomography (CT) is considered the standard for evaluating the anatomical conditions, determining the extent of pathological processes in the paranasal sinuses, and treatment planning [3]. A CT scan is performed as part of the preoperative diagnostic workup in patients with CRS. In contrast, follow-up examinations after surgery or during conservative treatment of acute sinusitis are rare.

Ultrasound (US) examination techniques are assumed to be useful in the diagnostic workup of PNS disease.

However, few evidence-based data currently exist on the value of sonography in various diseases of the PNS. In particular, mostly monocenter studies have shown different and sometimes contradictory results. Studies evaluating sonographic imaging compared US findings with CT images, others with MRI data, or with conventional radiographic overview images. In addition, many studies showed a highly heterogeneous patient population, including patients with both acute and chronic rhinosinusitis. Furthermore, meta-analyses have repeatedly pointed out the inadequate data quality [4,5,6,7,8,9,10].

There are currently only isolated observational studies comparing imaging modalities with intraoperative findings. For the first time, this prospective multicenter observational study compares the assessment of imaging modalities (B-scan US and CT scan) with the only valid imaging reference for assessing mucosal pathology, the intraoperative findings. The aim of the present work was to explore the potential and diagnostic value of ultrasound in CRS. In this context, the possibility of visualization and detection of mucosal swelling or secretion retention in the different PNS compartments using A- and B-scan US should be investigated. For the detection of mucosal changes, ultrasound and CT findings were compared with the intraoperative findings. In this way, the sensitivity, specificity, and diagnostic accuracy of each examination modality should be determined and validated by comparing the results from the different centers with high US expertise.

## 2. Materials and Methods

### 2.1. Participating Centers

The study was conducted as a multicenter observational investigation to minimize investigator-related bias.

All investigators had decades of experience in both sonographic and CT diagnosis of the PNS and conservative as well as surgical therapy of PNS diseases. They regularly perform PNS US in patients with CRS disease. All investigators were briefed in detail prior to the beginning of the study concerning the grading of the disease extent in US, CT scan, and surgery. Preoperative CT findings (point score (0–2)) were evaluated and modified according to Lund et al. [2] with the parameters “ventilated”, “partial opacification”, and “complete opacification” being used for evaluation. The maxillary sinus, the ethmoid, and the frontal sinus were scored separately with point values. US findings were evaluated according to the following aspects: “negative” for visualization of the soft tissue over the anterior sinus wall and the bony anterior sinus wall without other echoes, “mucosal swelling” on visualization of the soft tissue over the anterior sinus wall, the bony anterior sinus wall, and a widening of the epithelial layers behind it to more than 2 mm, “secretion retention” on visualization of the soft tissue over the anterior sinus wall, the bony anterior sinus wall, and a hyperechoic structure corresponding to the posterior sinus wall. Evaluation of the intraoperative findings was categorized with a score of 0 for morphologically unremarkable mucosa, a score of 1 for thickened mucosa, and a score of 2 for polyp-like changes or secretion retention applied for the maxillary sinus, the ethmoid sinus, and the frontal sinus, respectively (Table 1).

### 2.2. Inclusion Criteria

Patients were recruited over a period of 4 years.

The following inclusion criteria were mandatory:

1. patient age over 18 years and patients with chronic maxillary and/or frontal sinusitis who underwent CT of the PNS in anticipation of the need for surgical treatment.
2. B-mode US of the PNS as well as CT of the PNS preoperatively within 8 weeks, with continued A-mode US if available.
3. optional criteria: surgery no more than 2 days after CT and US; no systemic antibiotic or corticosteroid treatment between CT/US and surgery.

### 2.3. Exclusion Criteria

Patients excluded from the observational study were:

1. under 18 years of age
2. presence of a disease of the PNS other than CRS (e.g., tumor lesions)
3. pre-treatment with systemic corticosteroid or antibiotics within 10 days prior to the time of CT and/or US

Participation in the study was voluntary for the patients and based on informed and written consent. The provisions of the Declaration of Helsinki were met. Positive ethics votes from the relevant state medical associations were available for study inclusion.

### 2.4. Clinical Symptoms

Questionnaires were modified based on SNOT-20 scoring [11]. Out of all included patients, 117 reported nasal obstruction as a “major symptom”, and 105 reported nasal secretion. “Minor symptoms” were reported by 70 patients. Bronchial asthma was present in 26 patients.

### 2.5. Pre- and Intraoperative Examinations

#### 2.5.1. CT Evaluation

**Figure 1:**
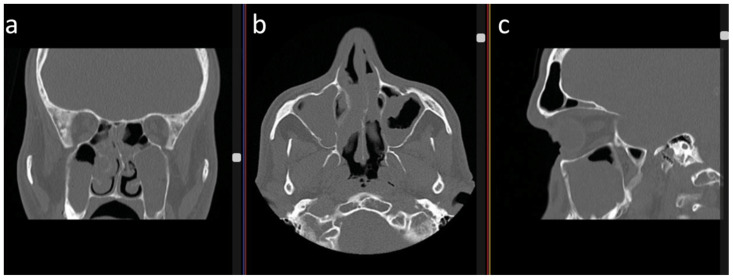
CT scan in three spatial planes in a patient suffering from CRS. (**a**) Coronal reconstruction with opacification of the maxillary sinus and the ethmoid sinus bilaterally on both sides. (**b**) Axial reconstruction; when viewed together with A, it can be observed that there is a subtotal obstruction in both maxillary sinuses due to secretion and mucosal swelling. (**c**) Sagittal reconstruction; the frontal sinus is largely unaffected. There is discrete residual ventilation in the cranial part of the maxillary sinus.

The CT examination (Figure 1) was evaluated preoperatively according to the guidelines described by Lund-Mackay [2]. We defined partial opacification as thickening of the affected sinus wall of more than 2 mm (Table 1).

#### 2.5.2. Evaluation of B-Mode US

The results of B-mode US were classified via point score (0–2), modified according to Hilbert 2001 [10]; Lichtenstein [12] (Table 1).

#### 2.5.3. Assessment of Intraoperative Findings

**Figure 2:**
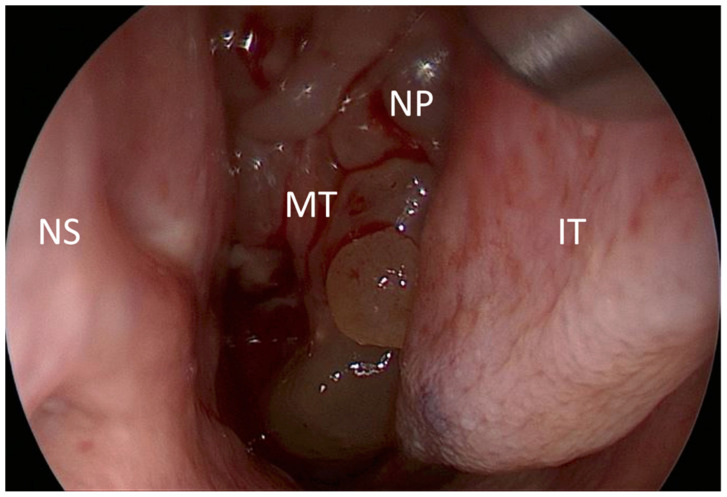
Intraoperative endoscopic view during paranasal sinus surgery in CRS. Nasal polyps (NP) embracing the middle turbinate (MT) and the inferior turbinate (IT) and reaching the nasal septum (NS).

**Figure 3:**
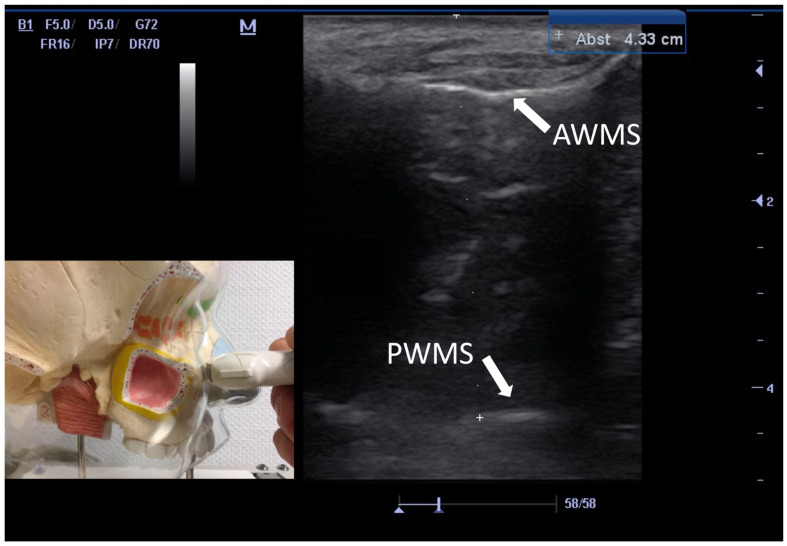
B-scan US of corresponding maxillary sinus from Figure 2. The osseus anterior wall of the maxillary sinus (AWMS) is identified at 1 cm depth. At a depth of 4.33 cm, the backscatter echo is depicted as hyperechoic back wall reflex (PWMS = posterior wall of the maxillary sinus).

In all surgically treated patients, intraoperative findings (Figure 2) were evaluated analogously to CT and US (Figure 3) assessment (Table 1).

### 2.6. Methods of A- and B-Mode US

Performance of B-mode sonography for each patient included in the study was mandatory, whereas A-mode imaging was optional. All participating centers used commercially available and certified US systems.

For A-mode US: A transducer with a nominal frequency between 3.5 MHz and 5 MHz and a penetration depth of at least 5 cm was applied.

B-mode: A linear transducer with one or more nominal frequencies between 5 MHz and 12 MHz and a penetration depth of at least 6 cm was applied.

**Figure 4:**
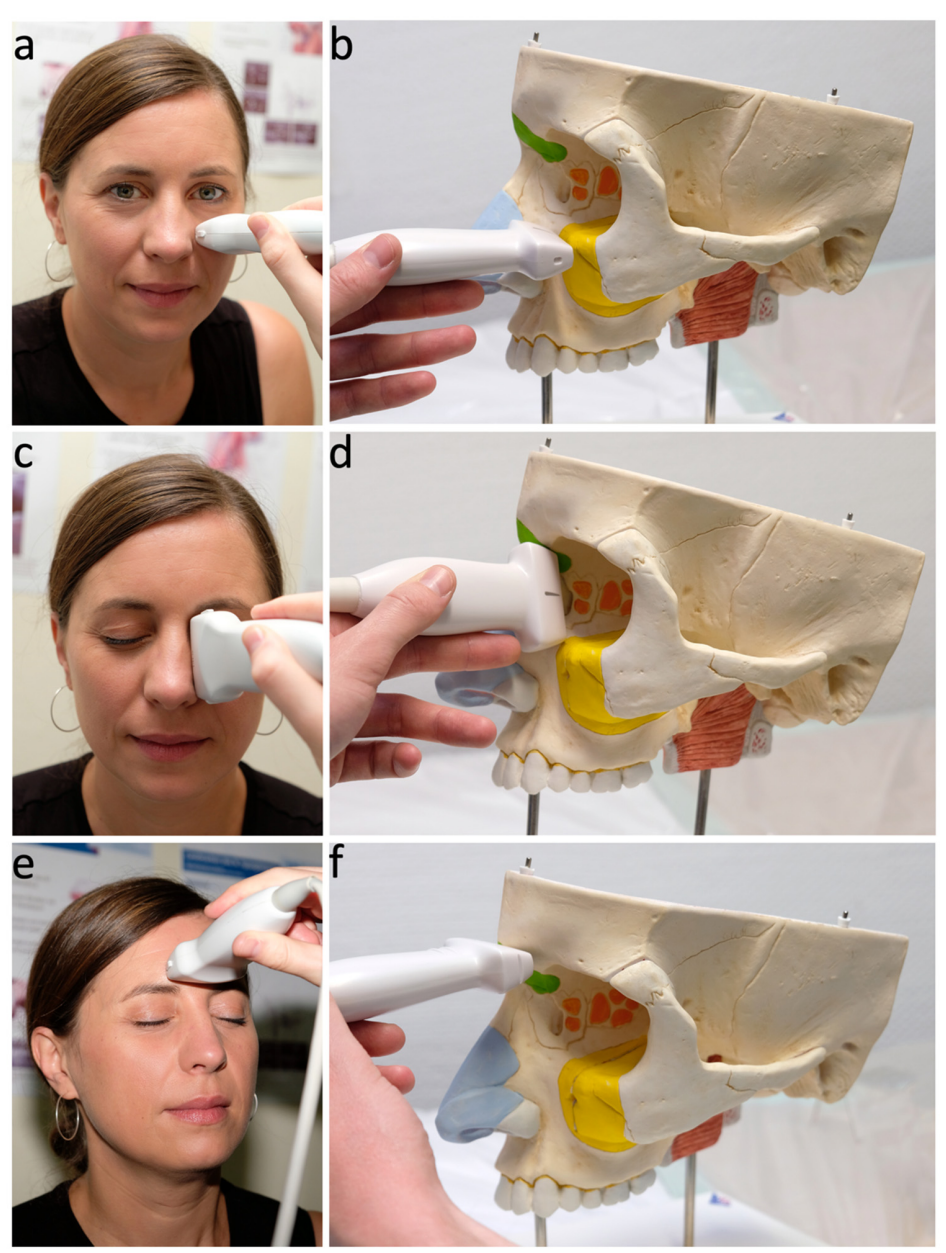
Transducer-positions in B-mode ultrasound of the paranasal sinuses. (**a**,**b**): Examination of the maxillary sinus in the axial plane. The transducer is positioned horizontally below the orbit on the anterior wall of the maxillary sinus. (**c**,**d**): Examination of the ethmoid sinus in a parasagittal plane. The transducer is positioned vertically in the medial corner of the eye following the course of the medial orbital wall. (**e**,**f**): Examination of the frontal sinus in the axial plane. The transducer is positioned horizontally in the lower midline of the forehead.

Patients were examined in an upright position with the head bent forward 30°. The anatomical region was documented for each US examination according to the following sonographic planes [13] (Figure 4).

### 2.7. Statistical Analysis

Sample size calculation was performed using commercially available PASS 2019 software (NCSS, LLC, Kaysville, UT, USA) with a statistical power of ß = 0.8 and a significance level of α = 0.05. Power analysis criteria were met with a minimum sample size of 110 patients.

The data were collected anonymously at each participating center. Subsequently, the anonymized data were transferred to the principal investigator for central statistical analysis. From the collected findings, the sensitivity, specificity, and diagnostic significance of CT and US were calculated. Included was a side-specific analysis for maxillary sinus, ethmoid sinus and frontal sinus. Imaging assessment was correlated with the extent of disease observed during paranasal sinus surgery.

## 3. Results

### 3.1. Patient Characteristics and US Examination

A total of 127 patients from 6 centers were included in the study; 67 male and 60 female patients were examined. The mean patient age was 66 years. Twenty-four patients received an A- and a B-scan examination, and 95 (75%) only a B-scan US. A complete CT scan data set was available in 120 cases (Figure 1). Because of the low response rate of optional A-mode sonography, it was not included in further analysis.

Surgery was conducted less than 4 weeks after imaging in 18 (14%) of the included patients.

### 3.2. Clinical Symptoms

Questionnaires were modified according to SNOT-20 scoring. Of the study patients, 117 reported nasal obstruction as a “major symptom”, 105 reported nasal secretion, and 70 patients reported minor symptoms. Bronchial asthma was present in 26 patients.

Comparison of intraoperative pathology assessment with B-mode US and CT imaging revealed a sensitivity of B-scan US of 89% and a specificity of 75% for correctly classifying sinus disease. CT had a sensitivity of 97% and specificity of 67% compared with the intraoperative findings (Table 2).

### 3.3. Comparison of the Anatomical Subregion 

In the subgroup analysis of the different anatomical regions, i.e., ethmoid, frontal and maxillary sinus, all imaging modalities were able to achieve a high level of agreement with the intraoperative findings in the assessment of the maxillary sinus (Table 3).

CT-scans showed the highest overall sensitivity in the assessment of the frontal, maxillary and ethmoid sinus, of 95%, 96% and 93%, respectively.

The assessment values of B-scan US were significantly lower, with sensitivities of 88% for the maxillary sinus, 53% for the ethmoid sinus and 45% for the frontal sinus (Table 3). No significant differences were seen in the side-separated assessment of the PNS for all imaging modalities compared to findings in surgery.

### 3.4. Comparison of Recruiting Centers 

When comparing the six centers, only B-scan sonography results were used due to the low number of A-scan findings. In three centers, there was high level of agreement in the assessment of PNS pathological findings with CT and US correlating with the intraoperative findings. However, the remaining centers noted discrepancies when comparing pre-operative imaging (US and CT) with the actual intraoperative situation. The highest sensitivity of all procedures and anatomic regions was observed for assessing the maxillary sinus (Table 4).

## 4. Discussion

CRS affects quality of life and presents significant long-term challenges to national health care systems. Estimates put the financial burden in the United States at USD 30 billion per year [14]. Thus, early diagnosis of CRS and initiation of therapy are not only critical for health economic reasons, but also directly associated with the patient’s quality of life. The presence of the two main symptoms “nasal obstruction” and “facial pain” plays an essential role in the diagnosis of CRS [1] and could be confirmed in the patients of our collective.

Symptoms and nasal endoscopy are the defining factors in the diagnosis of chronic diseases of the paranasal sinuses [15]. However, nasal endoscopy cannot reliably exclude chronic sinusitis in some cases. Therefore, imaging is necessary to confirm the suspicion of CRS, especially in prolonged and complicated courses. CT has established itself as the imaging modality of choice in this regard [1,3]. US is usually a rapidly available, radiation-free procedure for the patient. Despite their distribution in some countries, evidence-based data on US of the paranasal sinuses are sparse and heterogeneous. The aim of the present study was to explore the potential value of ultrasound in CRS compared to CT scan and surgery.

### 4.1. Diagnostic Utility of CT Scan in CRS

CT of the paranasal sinuses represents the imaging modality of choice for the diagnosis of CRS. The depiction of anatomical features improves surgical planning and, therefore, patient safety. Our results confirm a high sensitivity (97%) and specificity (67%) of CT when compared with intraoperative findings. These data show the good reliability of this diagnostic procedure already reported in the literature [16] and underline the importance of CT in the context of preoperative imaging. If CT is inconspicuous and symptoms persist, further differential diagnostic considerations are mandatory [17]. However, CT must be interpreted in conjunction with clinical symptoms and endoscopic findings, as CT of the paranasal sinuses reveals incidental mucosal abnormalities in approximately 41% of asymptomatic patients [18,19].

### 4.2. Value of US in the Diagnosis of CRS

Zagolski et al. reported concordances between CT findings and US in maxillary sinus disease in 81.4% of cases and frontal sinus disease in 75% of cases [8]. Similar observations can be found in the literature [4,12,16,20,21].

We confirmed these findings for the maxillary sinus by comparing B-scan US and CT scan (Table 3). Other investigators found little agreement between US, CT, and radiographic findings. It should be noted that a major limitation of the above studies is that the US examination was compared only with the corresponding “imaging standard” available at the time. Endoscopic findings during surgery were not considered in the assessment. For this reason, the present work should improve the comparability of available diagnostic procedures.

### 4.3. A-Scan US

Because A-scan US was used in only 24 cases, this imaging method was excluded from the statistical evaluation. In our view, this corroborates the low relevance of A-mode sonography in a clinical setting. In the available literature, A-mode ultrasound is considered inadequate compared to CT [11,22]. Especially in chronic sinusitis, it probably may not be recommended due to its susceptibility to artifacts and potential operator dependency [19,23,24,25,26,27,28].

### 4.4. B-Mode US

Several studies concerning paranasal sinus imaging in acute sinusitis have been performed, often with substantial discrepancies regarding patient selection and technical implementation.

Nevertheless, so far, fair to good agreement between B-mode US of the maxillary sinuses and the corresponding reference imaging has been reported [4]. Hilbert et al. studied a collective of 50 ventilated patients with B-mode US. The authors correlated their US findings with CT results and described a sensitivity of 100% and a specificity of 97% [20]. Lichtenstein et al. recommended US of the maxillary sinus as the diagnostic tool of choice in sinusitis with a sensitivity of 67% and a specificity of 87% [12]. Notably, his findings were published in 1989, when a CT scan was not ubiquitously available or even recommended.

In a prospective study, Gianoli et al. compared PNS findings obtained by B-mode US with CT findings of 41 patients. The authors described a sensitivity of B-mode US for each of the sub-localizations of 100% with a specificity of 98% for the maxillary sinus, 100% for the frontal sinus, and 94% for the ethmoid sinus [5].

Tiedjen et al. could not reproduce these numbers. They described sensitivity of B-scan US of 72.8% regarding pathologies of the maxillary sinus, 23.1% for the frontal sinus and 11.3% for the ethmoid sinus. In contrast, specificity was significantly higher, with 98% for the maxillary sinus, 100% for the frontal sinus and 99% for the ethmoid sinus [6].

Furthermore, bias due to study design must be considered when interpreting these findings. Nevertheless, the latter observations concur with our own experiences even in the investigated sub-localizations.

For our collective, CT vs. B-scan US showed comparable results (Table 2). High values were found for B-mode US, with a sensitivity of 89% and specificity of 75%. This was achieved for the maxillary sinus with a sensitivity of 83% in our collective. However, due to the susceptibility to artifacts, visualization of changes in the anterior ethmoid and the frontal sinus via US are expectedly less reliable. Comparison between US and CT revealed a sensitivity of less than 60% for these regions.

When comparing centers and PNS assessment by CT, the assessments showed high sensitivity (84–100%). These results underscore the high degree of standardization in the interpretation and evaluation regarding CT scans. In contrast, the sonographic assessment of the centers was heterogeneous, with sensitivities ranging from 20% to 100%. The maxillary sinus was correctly assessed with the highest sensitivity ranging from 50% to 100%. The ethmoid sinus and the frontal sinus region exceeded values of more than 50% in only 4 cases. Due to technical limitations, it appears hardly possible to determine by US whether a frontal sinus shows thickened epithelium in the posterior wall. Carefully interpreting our data, inferiority of US assessment regarding ethmoid sinus and frontal sinus leads us to recommend US for assessment of the maxillary sinus only. Some limitations of our study have to be pointed out. The variability of the results with respect to the anatomical region might be due to basic physical properties or the dependence of the respective procedure on the examiner. Other technical difficulties, such as the detection of partial opacification of the maxillary or frontal sinuses that are not reached by the sound wave, as well as differences regarding the occurrence of posterior wall echoes due to variations in the transducer position or head position of the patient, are characteristic pitfalls in US of the PNS [3].

These aspects must also be taken into account in the assessment of our collective. Despite meticulous briefing, we noted differences in the participating centers. These factors apparently resulted in a lower diagnostic impact in the evaluation of US as opposed to CT scan, combined with a higher intra- and interobserver variability.

## 5. Conclusions

We present the first prospective multicenter study comparing sonographic techniques and CT scans of the paranasal sinuses with the intraoperative findings in CRS. Surgery was defined as the reference for all imaging results and subsequently compared to both imaging modalities in assessing the pathology. On the basis of the available data set, we were not able to assess the value of A-scan US. Based on the data available in the literature, B-scan US should be the ultrasound method of choice in a clinical setting. An investigation of the potential value of A-scan US would require another study, probably in an outpatient setting.

Computed tomography demonstrated the highest concordance with the intraoperative findings. In our study, an interval of 8 weeks between imaging and surgery did not confound CT or US findings. Particularly for the frontal sinus and the ethmoid sinus, the validity of CT was significantly higher than that of B-scan US. Despite the high expertise of all investigators and a standardized examination protocol, interindividual operator variations and US image interpretations were potentially responsible for the inferior assessment of CRS with US.

The good concordance between preoperative CT pathologic assessment and intraoperative findings confirm this procedure for CRS diagnosis and workup. US of the paranasal sinuses is applicable in everyday clinical practice, but should be reserved for specific questions or conditions. In the hands of the experienced investigator, it may serve as a complementary radiation-free screening procedure for acute complications of PNS [29] or in cases of mid-face trauma [30]. In conclusion, our data show a high degree of comparability with other recent studies [31,32]. The fast availability and the possibility of direct correlation with endoscopy make ultrasound a promising complementary diagnostic tool in the context of CRS. This potential needs to be further explored. Finally, the rapid technical progress (e.g., fusion imaging, machine learning) might enhance the diagnostic spectrum in PNS disease and imaging. Whether US can constitute a standard of imaging modality in chronic PNS disease remains to be seen.

## Figures and Tables

**Table 1:** Evaluation of preoperative CT scan, preoperative ultrasound, and findings in surgery.

		Right			Left	
CT parameters	ventilated	partial opacification	complete opacification	ventilated	partial opacification	complete opacification
Ultrasound parameters	negative	mucosal swelling	secretion retention	negative	mucosal swelling	secretion retention
Intraoperative parameters	negative	mucosal thickening	polypoid tissue or secretion retention	negative	mucosal thickening	polypoid tissue or secretion retention
ethmoid sinus	0	1	2	0	1	2
maxillary sinus	0	1	2	0	1	2
frontal sinus	0	1	2	0	1	2

**Table 2:** Comparison of intraoperative pathology assessment B-mode US and CT imaging findings.

No. of Cases	Sensitivity [95% CI]	Specificity [95% CI]
**B-mode-sonography vs. intraoperative pathology**		
119	0.89 [0.81; 0.94]	0.75 [0.19; 0.99]
**Computed tomography vs. intraoperative pathology**		
120	0.97 [0.93; 0.99]	0.67 [0.22; 0.96]

Sensitivity and specificity of sonography and CT scan compared with findings obtained during surgery. The 95% confidence interval (CI) is given in square brackets.

**Table 3:** Subgroup analysis of US, CT scan and intraoperative pathology assessment considering the anatomical subregions of ethmoid, frontal and maxillary sinus.

	*n*	Sensitivity [95% CI]	Specificity [95% CI]
	**B-mode-sonography vs. intraoperative pathology**		
ethmoid sinus	115	0.53 [0.43; 0.63]	0.75 [0.43; 0.95]
maxillary sinus	121	0.88 [0.80; 0.93]	0.80 [0.52; 0.96]
frontal sinus	117	0.45 [0.33; 0.58]	0.92 [0.81; 0.98]
	**Computed tomography vs. intraoperative pathology**		
ethmoid sinus	118	0.93 [0.87; 0.97]	0.64 [0.35; 0.87]
maxillary sinus	119	0.96 [0.90; 0.99]	0.53 [0.27; 0.79]
frontal sinus	115	0.95 [0.87; 0.99]	0.80 [0.66; 0.90]

Sensitivity and specificity of sonography and CT scan compared with findings obtained during surgery. A partial mucosal swelling or secretion retention was defined as a positive finding. The 95% confidence interval (CI) is given in square brackets.

**Table 4:** Center comparison of B-scan US and CT assessment of pathology compared to the intraoperative findings.

	Center 1	Center 2	Center 3	Center 4	Center 5	Center 6
	**Sensitivity of B-mode-sonography vs. intraoperative pathology [95% CI]**					
ethmoid sinus	0.56 [0.30; 0.80]	0.20 [0.04; 0.48]	0.82 [0.48; 0.98]	0.42 [0.20; 0.67]	0.74 [0.49; 0.91]	0.27 [0.06; 0.61]
maxillary sinus	1.00 [0.81; 1.00]	0.50 [0.23; 0.77]	0.69 [0.39; 0.91]	0.95 [0.74; 1.00]	0.95 [0.74; 1.00]	1.00 [0.72; 1.00]
frontal sinus	0.38 [0.14; 0.68]	0.00 [0.00; 0.97]	0.43 [0.10; 0.82]	0.46 [0.19; 0.75]	0.50 [0.25; 0.75]	0.33 [0.07; 0.70]
	**Sensitivity of computed tomography vs. intraoperative pathology [95% CI]**					
ethmoid sinus	1.00 [0.77; 1.00]	0.93 [0.68; 1.00]	1.00 [0.77; 1.00]	0.84 [0.60; 0.97]	0.95 [0.74; 1.00]	1.00 [0.72; 1.00]
maxillary sinus	1.00 [0.79; 1.00]	1.00 [0.77; 1.00]	1.00 [0.75; 1.00]	0.89 [0.67; 0.99]	1.00 [0.82; 1.00]	1.00 [0.72; 1.00]
frontal sinus	0.92 [0.62; 1.00]	1.00 [0.03; 1.00]	1.00 [0.59; 1.00]	0.85 [0.55; 0.98]	1.00 [0.79; 1.00]	1.00 [0.66; 1.00]

Sensitivity of sonography and CT scan compared with findings obtained during surgery. The 95% confidence interval (CI) is given in square brackets.

## Data Availability

The data presented in this study are available on request from the corresponding author.
